# Anterior skull base reconstruction using nasoseptal flap: cadaveric feasibility study and clinical implication [SevEN-001]

**DOI:** 10.1186/s40463-020-00460-3

**Published:** 2020-09-21

**Authors:** Kyoung Su Sung, Jaejoon Lim, Minkyun Na, Sanghun Lee, Ju-Seong Kim, Je Beom Hong, Chang-Ki Hong, Ju Hyung Moon

**Affiliations:** 1 Department of Neurosurgery, Dong-A University Hospital, Dong-A University College of Medicine, Busan, Republic of Korea; 2 Department of Neurosurgery, Bundang CHA Medical Center, CHA University College of Medicine, Seongnam, Republic of Korea; 3 Department of Neurosurgery, Hanyang University Seoul Hospital, Hanyang University College of Medicine, Seoul, Republic of Korea; 4 Department of Neurosurgery, Cheonan Chungmu Hospital, Cheonan, Republic of Korea; 5 Department of Neurosurgery, Ewha Womans Seoul Hospital, Ewha Womans University College of Medicine, Seoul, Republic of Korea; 6 Department of Neurosurgery, Kangbuk Samsung Hospital, Sungkyunkwan University School of Medicine, Seoul, Republic of Korea; 7 Department of Neurosurgery, Brain Tumor Center, Gangnam Severance Hospital, Yonsei University College of Medicine, Seoul, Republic of Korea; 8 Department of Neurosurgery, Brain Tumor Center, Severance Hospital, Yonsei University College of Medicine|, 50-1 Yonsei-ro, Seodaemun-gu, Seoul, 03772 Republic of Korea

**Keywords:** Anterior skull base, Nasoseptal flap, Sphenoidotomy, Skull base reconstruction

## Abstract

**Background:**

Pedicled nasoseptal flap (PNSF) has significantly improved the surgical outcomes of endoscopic endonasal approach (EEAs) by reducing cerebrospinal fluid (CSF) leakage. The purpose of this study is to assess the feasibility of using a PNSF for anterior skull base (ASB) reconstruction and to describe a method to compensate for a short flap based on our results.

**Methods:**

In this cadaveric study, ASB dissection without sphenoidotomy was performed using 10 formalin-fixed and 5 fresh adult cadaver specimens, and the sufficiency of the PNSF to cover the ASB was assessed. After the sphenoidotomy, the length by which the PNSF fell short in providing coverage at the posterior wall of the frontal sinus (CPFS), and the extent of the anterior coverage from the limbus (CL) of the sphenoid bone was measured.

**Results:**

Without sphenoidotomy, the mean length of the remaining PNSF after the coverage of the posterior wall of the frontal sinus was 0.67 cm. After sphenoidotomy, the PNSF fell short by a mean length of 2.10 cm, in providing CPFS. The CL was 1.86 cm. Based on these findings, defects resulting from an endoscopic resection of ASB tumors were reconstructed using PNSF without total sphenoidotomy in 3 patients. There were no postoperative CSF leaks or complications.

**Conclusions:**

The use of PNSF for ASB reconstruction may be insufficient to cover the entire ASB defect after removal of large lesions which need total sphenoidotomy. When possible, by leaving some portion of the anterior sphenoid wall for supporting the PNSF, successful ASB reconstruction could be achieved in endoscopic resection of ASB tumors. Additional methods might be needed in some cases of large ASB lesions wherein the anterior sphenoid wall should be removed totally and the ASB defect is too large.

## Background

After the development of the pedicled nasoseptal flap (PNSF), a breakthrough method to prevent the cerebrospinal fluid (CSF) leakage, there have been advances in the endoscopic endonasal approaches (EEAs) extending to the treatment of entire skull base lesions [1, 2]. However, due to a limitation in size and the arc of rotation, the PNSF can be less successful in covering the entire affected area. For lesions in the anterior skull base (ASB), the routine PNSF occasionally could be short, leading to postoperative CSF leakage. Because of these reasons, several techniques, such as the slit incision, lengthening the PNSF with extended dissection into the pterygopalatine fossa, and extended flap, have been reported with successful results [3–6]. Despite these modifications, the ASB reconstruction using the PNSF can be unsuccessful because of not enough extension of length or the impairment of PNSF rigidity such as decreased vascular supply. Extensive manipulation of nasal mucosa in some methods can also cause nasal complications and decreased nasal function.

The theoretical anterior limit of the ASB is the posterior wall of the frontal sinus [7]. Some studies on the feasibility of using a PNSF for ASB reconstruction have measured the distance from the anterior border of the planum sphenoidale to the posterior wall of the frontal recess [8, 9]. These studies were based on the assumption that the ASB defect is not as large as to perform the sphenoidotomy. However, the preoperative plan for lesions requiring additional removal of the planum sphenoidale and tuberculum sellae should be different from the EEAs for lesions limited to the cribriform plate, which is also an important issue to consider when using PNSF for reconstruction. Therefore, a study on the range of coverage provided by the PNSF in cases of ASB defects with or without sphenoidotomy is likely to provide meaningful results. We in this study have described some clinical cases and a method to compensate for the short in flap length.

## Material and methods

### Anatomical study

This anatomical study was performed at the Surgical Anatomy Laboratory of the Yonsei University College of Medicine and Severance hospital. Five fresh and 10 formalin-fixed cadaver specimens were used in this study. All the cadavers were of Koreans who were over 18 years of age. The exclusion criteria included a history of ASB mass, gross sinonasal pathology, prior sinonasal and ASB surgery, maxillofacial trauma, and anatomical abnormalities. Our study included sphenoid sinuses of the presella (*n* = 1) and sella (*n* = 14) types. Endonasal anatomical dissections were carried out using rod lens endoscopes (Karl Storz Endoscopy-America, Inc., Culver City, CA, and Stryker Neuroendoscopy, 2825 Airview Boulevard, Kalamazoo, MI 49002 U.S.A) attached to a camera with a charge-coupled device sensor. All cadaver dissections and measurements were performed under a navigation system (Surgical Navigation System, Medtronics, Minneapolis, MN, USA, and Stryker Navigation System, 2825 Airview Boulevard, Kalamazoo, MI 49002 U.S.A).

**Fig. 1:**
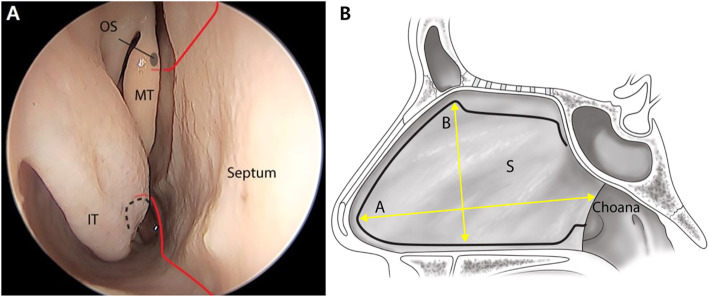
Schematic diagrams illustrating the dissection procedures. **a** Endoscopic view of right nostril. **b** Sagittal view of the pedicled nasoseptal flap. OS: sphenoid ostium, MT: middle turbinate, IT: inferior turbinate, S: septum

The PNSF was prepared by an EEA through the right-side in all 15 cadavers. Under visualization with a 0-degree endoscope, the cut started from the midpoint at the superior margin of the choana and extended along the posterior margin of the vomer toward the nasal floor (Fig. 1a). The inferior cut proceeded anteriorly along the junction between the septum and the nasal floor to the caudal margin of the septum. Next, a hemitransfixion incision was made along the caudal border of the septum. From the superior portion of the vertical cut, the superior cut proceeded posteriorly along the nasal dorsum to the attachment of the middle turbinate. Further, the incision was continued for about 1 cm inferiorly to the nasal vault to protect the olfactory area. The cut proceeded in parallel to the skull base and ended at the sphenoid ostium (Fig. 1b). The PNSF was detached from the septum by a subperichondrial and subperiosteal dissection. The posterior septal artery was preserved for the PNSF. The pedicle of the PNSF was transected to measure the size of the PNSF. The longest length from the anterior septal angle to the pedicle of the posterior septal artery was termed as “A,” and the height of the PNSF was termed as “B” (Fig. 1b, Table 1).

**Table 1:** Measurements of the anterior skull base and nasoseptal flap

No.	Without sphenoidotomy (cm)	With sphenoidotomy (cm)		PNSF Length (cm)		ASBD (cm)
	CPFS	CPFS	CL	A	B	
**1**	1.5	- 2.2	1.8	6.9	4.6	4.0
**2**	0.7	- 2.3	1.5	5.6	4.1	3.8
**3**	0.9	- 1.9	2.2	6.4	3.9	4.1
**4**	1.3	- 2.7	1.5	6.3	3.7	4.2
**5**	0.2	- 2.1	2.4	5.9	4.0	4.5
**6**	0.7	- 2.0	1.7	5.4	3.7	3.7
**7**	0.3	- 1.7	2.0	6.0	4.0	3.7
**8**	0.4	- 2.4	1.4	5.6	3.6	3.8
**9**	0.9	- 0.9	3.3	6.5	3.8	4.1
**10**	0.3	- 2.3	1.5	6.0	3.7	3.8
**11**	0.6	- 2.2	1.8	6.4	3.5	4
**12**	0.8	- 2.2	1.5	5.9	4	3.7
**13**	0.2	- 1.9	1.3	5.8	3.5	3.2
**14**	0.8	- 2.4	1.9	6.2	4	4.3
**15**	0.5	- 2.3	2.2	6.6	4.2	4.5
	0.67 (0.38) ^a^	- 2.10 (0.41) ^a^	1.86 (0.51) ^a^	6.10 (0.42) ^a^	3.88 (0.29) ^a^	3.96 (0.34)^a^

^a^Data are presented as mean cm (standard deviation)

*CPFS* Coverage at the posterior wall of the frontal sinus, *CL* Coverage from the limbus, *PNSF* Pedicled nasoseptal flap, *ASBD* Anterior skull base distance

**Fig. 2:**
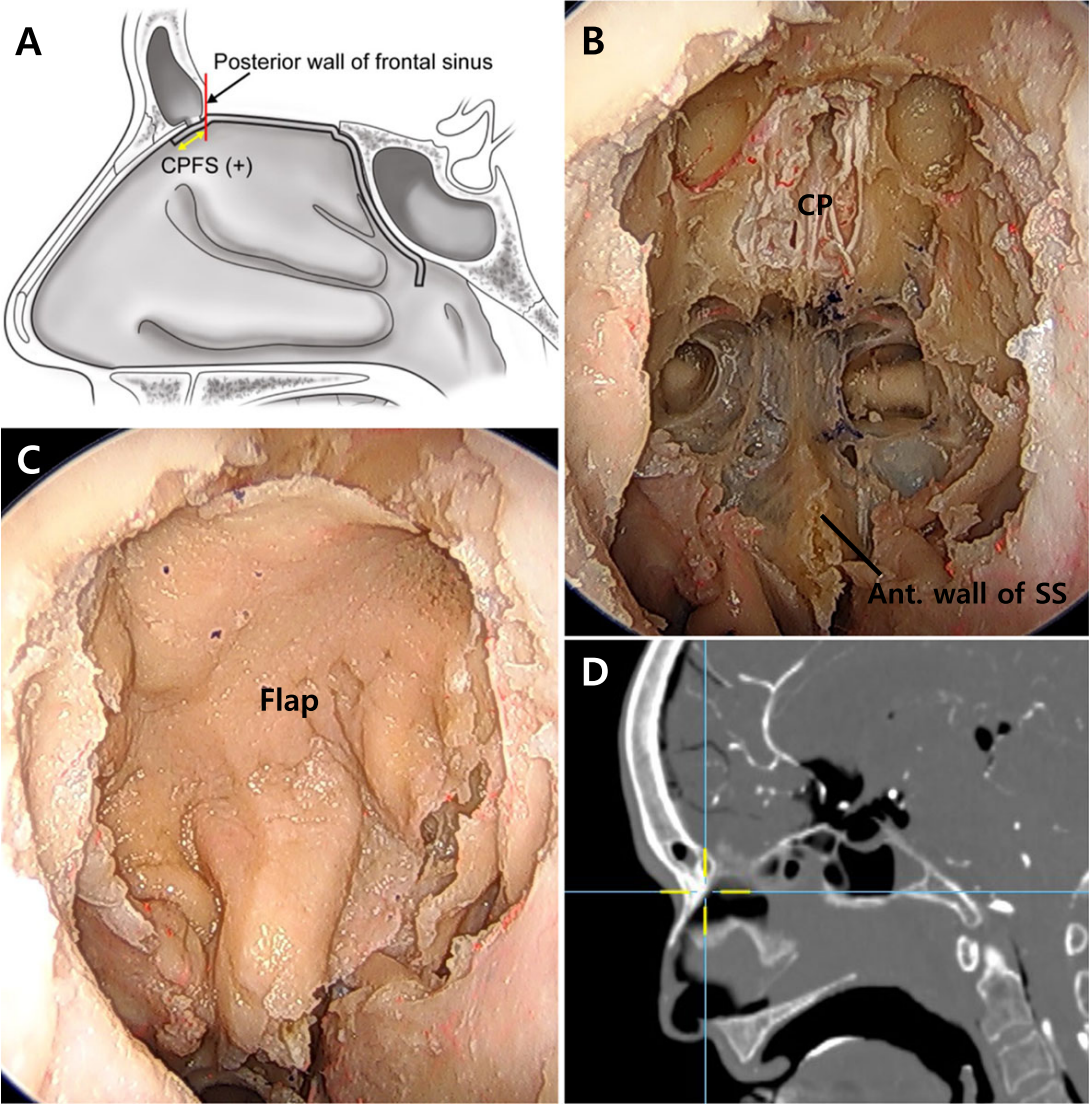
Anterior skull base reconstruction without sphenoidotomy. **a** An illustration of the sagittal view without sphenoidotomy. **b** A 0-degree endoscopic view of the anterior skull base without sphenoidotomy and (**c**) the view after covering with the pedicled nasoseptal flap. **d** Confirmation of the end point of the coverage under navigation. CPFS: coverage at the posterior wall of the frontal sinus, CP: cribriform plate, SS: sphenoid sinus

**Fig. 3:**
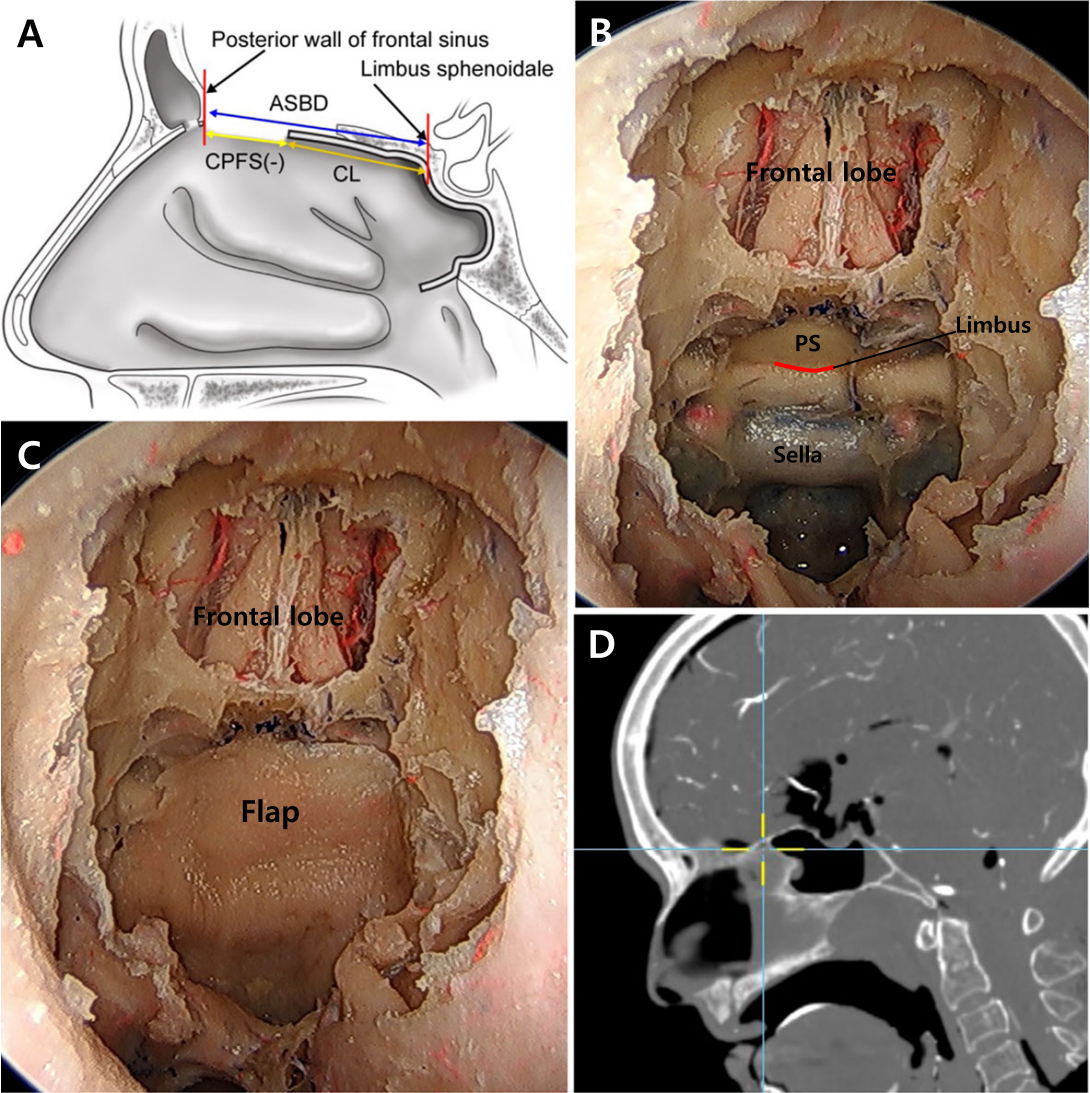
Anterior skull base reconstruction with sphenoidotomy. **a** An illustration of the sagittal view with sphenoidotomy. **b** A 0-degree endoscopic view of the anterior skull base with sphenoidotomy and (**c**) the view after covering with the pedicled nasoseptal flap. **d** Confirmation of the end point of the coverage under navigation. ASBD: anterior skull base distance, CPFS: coverage at the posterior wall of the frontal sinus, CL: coverage from the limbus, PS: planum sphenoidale

An EEA was used for ASB resection. We performed bilateral middle and superior turbinectomy. The superior part of the nasal septum was resected, and bilateral ethmoidectomy was performed. The anterior limit of the ASB was the posterior wall of the frontal recess. The posterior limit was the anterior wall of the sphenoid sinus. The lateral limit was the bilateral orbit. The opening of the frontal sinus was exposed, and the PNSF was placed to cover the ASB defect without traction. The remaining length of the PNSF from the posterior wall of the frontal sinus was measured (Fig. 2a, b, c). Subsequently, sphenoidotomy was performed. The sella floor, tuberculum sella, and the planum sphenoidale were exposed. The inferior portion of the anterior wall of the sphenoid sinus was drilled to release the tension in the PNSF, which was then placed anteriorly along the sella floor and tuberculum sella. The coverage range was measured from the limbus, and the insufficiency in coverage was measured from the posterior wall of the frontal sinus (Fig. 3a, b, c). After performing the measurements, we confirmed the coverage range of the PNSF using a navigation system (Fig. 2d and 3d).

The anterior skull base distance (ASBD) was defined as the length from the posterior wall of the frontal sinus to the limbus of the sphenoid bone. Coverage from the limbus (CL) of the sphenoid bone was defined as the length of coverage of the PNSF anteriorly from the limbus after performing sphenoidotomy. The coverage at the posterior wall of the frontal sinus (CPFS) was defined as the length of the PNSF anteriorly from the posterior wall of the frontal sinus. If the CPFS value was positive, the PNSF could reach and cover the posterior wall of the frontal sinus. However, if it was negative, the length of the PNSF was insufficient to reach the posterior wall of the frontal sinus. We have shown these measurements in Figs. 2a, 3a, and b.

### Clinical implications

Based on the anatomical details and results obtained from our cadaveric study, ASB reconstruction using PNSF without total sphenoidotomy was planned for 3 patients who had ASB defects after tumor resection by EEAs. We performed a retrospective review of the demographic, clinical, surgical, and outcome data of the patients selected to undergo skull base reconstruction using PNSF without total sphenoidotomy. This clinical study was approved by our Institutional Review Board, which waived the requirement for informed consent from the patient.

The preparation of the PNSF and reconstruction were performed by an EEA, in the same manner as in the cadavers. Once the flap was positioned correctly and it completely covered the defect, Bemsheets surgical pads (Kawamoto Bandage Material Co., Ltd., Osaka, Japan) soaked in an antibiotic ointment were packed over the entire surface of the flap overlapping the edges. This helped in holding the flap in place and in obliterating the dead space between the flap and the underlying tissue, ensuring that all aspects of the flap, including the pedicle, were in direct contact with the wider outer boundary of the bony defect. A polyvinyl alcohol (PVA) sponge (Merocel: Medtronic Xomed, Jacksonville, Florida, USA) covered with the finger portion of a surgical glove was inserted to exert indirect compression of the flap against the defect. This nasal tampon was left in place for 5 to 7 days. Silastic splints were placed over the exposed septal cartilage for approximately 2 weeks to protect the denuded septum and the lateral nasal wall during wound healing.

## Results

Table 1 shows the PNSF measurements and the other values associated with the ASB. In all 15 dissections without sphenoidotomy, the PNSF provided sufficient coverage, reaching the posterior wall of the frontal sinus without over traction. The mean length of the PNSF that was in excess after providing CPFS was 0.68 ± 0.38 cm, although, in 6 of the 15 dissections (40%), it was ≤0.5 cm. However, when sphenoidotomy was performed, the PNSF could not reach the posterior wall of the frontal sinus in all dissections. The mean insufficiency in the length of the PNSF to the posterior wall of the frontal sinus was 2.10 ± 0.41 cm. The mean value of the CL was 1.86 ± 0.51 cm.

The difference in the CPFS with and without sphenoidotomy indicated the PNSF length needed to provide coverage from the clivus in the sphenoid sinus, sella floor, and tuberculum. The mean PNSF length required to cover this area was 2.77 ± 0.6 cm (Range: 1.8–3.7 cm). The 9th cadaver showed a presella type of sphenoid sinus, and the PNSF length required to cover this area in the 9th cadaver was 1.8 cm, which was the minimum value seen among all the dissections. In the PNSF measurements, while the mean value for “A” was 6.10 ± 0.42 cm, that for “B” was 3.88 ± 0.29 cm. The mean ASBD was 3.96 ± 0.34 cm.

### Brief illustrative cases

We performed a review of the medical records of three patients who underwent endoscopic skull base reconstruction using PNSF without total sphenoidotomy. The clinical data of these patients are summarized in Table 2. All flaps were viable after harvesting, with no signs of congestion or discoloration. Postoperative magnetic resonance imaging (MRI) showed that the PNSF without total sphenoidotomy provided full coverage of the ASB from the posterior frontal sinus wall to the posterior end of the cribriform plate, covering all surgical defects successfully. No perioperative lumbar drain was used in any of the patients. All the patients required daily nasal toilette and came to the outpatient clinic for debridement every week for 1 month after the operation, and every 2 weeks thereafter until mucosalization was complete. Consequently, all three patients healed uneventfully with no CSF leaks or any other postoperative complications.

**Table 2:** Clinical and demographic characteristics of patients using PNSF without sphenoidotomy

No.	Sex/Age (yrs)	Pathology	CSF leak area (leak flow)	Surgical approach	Sphenoidotomy	Ant. limit of coverage	Postop. CSF leak	Follow-up period
**1**	M/47	Olfactory Neuroblastoma	CP (high flow)	Endonasal	Partial	Post. wall of the FS	No	12 mos
**2**	F/43	Olfactory Neuroblastoma	CP (high flow)	Endonasal	Partial	Post. wall of the FS	No	9 mos
**3**	F/59	Meningioma	CP & FS (high flow)	Endonasal& Transcranial	None	Ant. wall of the FS	No	6 mos

*PNSF* Pedicled nasoseptal flap, *CSF* Cerebrospinal fluid, *CP* Cribriform plate, *FS* Frontal sinus, *Ant*. Anterior, *Post*. Posterior, *Postop*. Postoperative, *mos* Months

#### Case 1: recurrent olfactory neuroblastoma

**Fig. 4:**
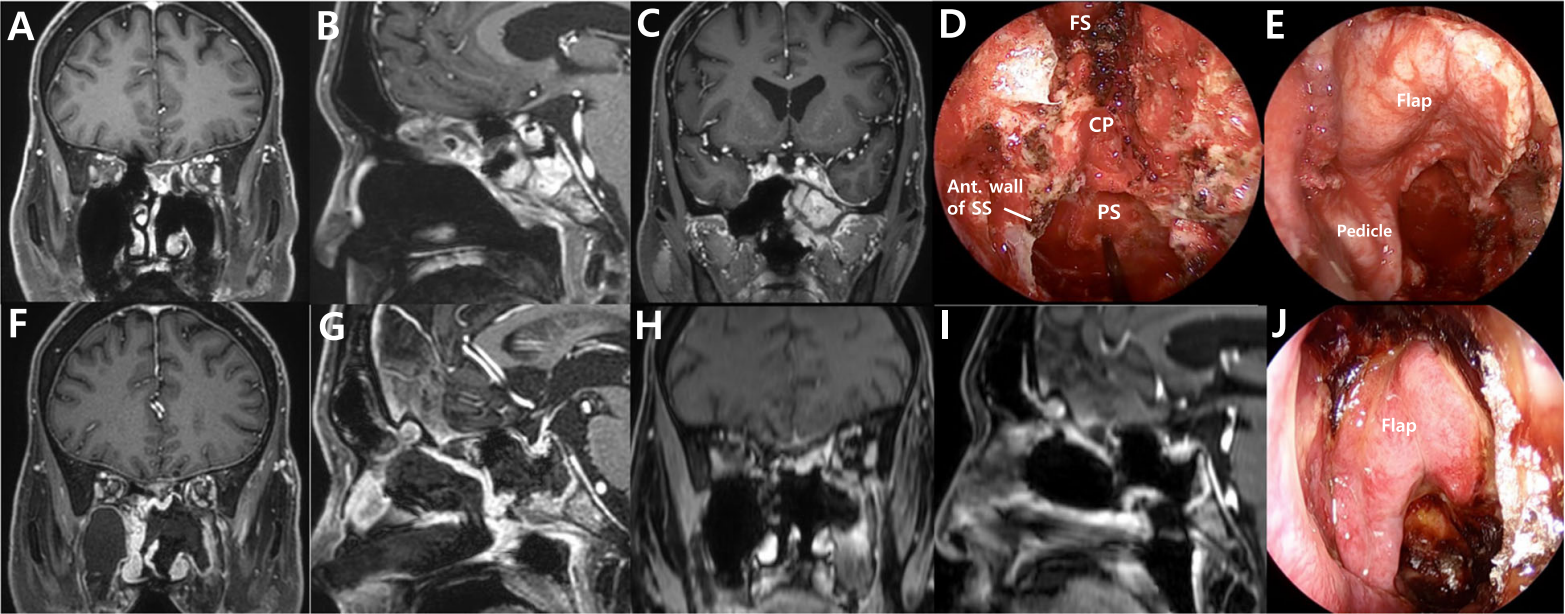
Images from Case 1. A recurrent olfactory neuroblastoma. **a** Coronal enhanced magnetic resonance imaging (MRI) reveals a recurrent tumor on the frontal base, which was operated upon by an endoscopic endonasal approach from the left nostril. **b** Enhanced sagittal MRI shows a mass with irregular margins in the left ethmoid sinus extending to the frontal base bone, dura, and sphenoid sinus. **c** The tumor can be seen extending into the left side of the sphenoid sinus and nasal cavity. **d** The right lateral side of the sphenoid sinus wall is left intact to support the pedicle of the PNSF. **e** The PNSF covers the ASB defect through the bony support of the remnant lateral side of the sphenoid sinus wall. **f**, **g** Postoperative coronal and sagittal MRI reveals a well enhanced PNSF and coverage of the ASB defect. **h**, **i** One year after the operation, the follow-up coronal and sagittal MRI showed well-healing state of the nasal mucosa and no recurrence of the tumor. **j** An endoscopic view shows a well-harvested PNSF at 2 weeks after the operation. FS: frontal sinus, CP: cribriform plate, SS: sphenoid sinus, PS: planum sphenoidale, PNSF: pedicled nasoseptal flap, ASB: anterior skull base

A 47-year-old man presented with recurrent olfactory neuroblastoma, which was operated upon by a left side EEA. Enhanced MRI revealed a mass with irregular margins in the left ethmoid sinus that extended to the frontal base bone, dura, and sphenoid sinus (Fig. 4a, b). Coronal MRI showed that the tumor extended into the left side of the sphenoid sinus (Fig. 4c). The tumor was resected, and the right lateral side of the sphenoid sinus wall was not removed to support the PNSF (Fig. 4d). The PNSF was used to cover the ASB defect through the bony support of the remnant lateral side of the sphenoid sinus wall (Fig. 4e). Postoperative coronal and sagittal MRI revealed well enhanced PNSF and coverage of the ASB defect (Fig. 4f, g). An endoscopic view 2 weeks after the operation showed a well-harvested PNSF (Fig. 4j). The follow-up MRI taken 1 year after operation showed stable disease status and good healing of nasal mucosa (Fig. 4h, i). No CSF leakage was encountered during the 1-year follow-up period.

#### Case 2: olfactory neuroblastoma

**Fig. 5:**
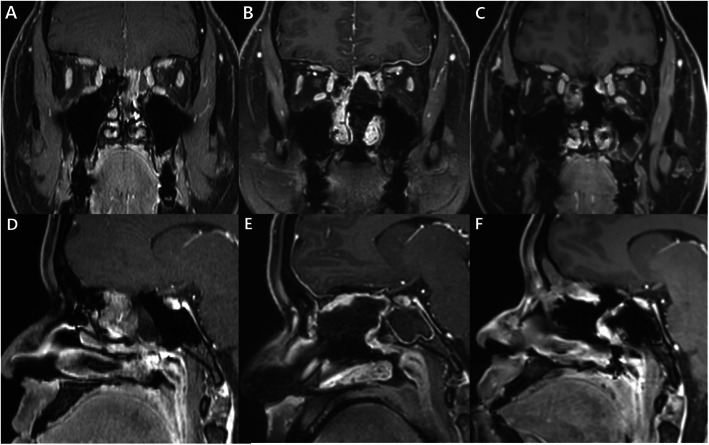
Images from Case 2. An olfactory neuroblastoma. **a** Enhanced coronal MRI reveals a mass with irregular margins on the left side of the upper nasal cavity extending to the frontal base. **d** The tumor can be seen invading the frontal base and extending to the anterior wall of the sphenoid sinus. **b**, **e** A well-enhanced PNSF and coverage state of the ASB defect can be seen in the postoperative coronal and sagittal MRI. **c**, **f** The follow-up coronal and sagittal MRI revealed stable disease status and well-healing state of the nasal mucosa 6 months after the operation. ASB: anterior skull base, PNSF: pedicled nasoseptal flap

A 43-year-old woman presented with nasal stiffness and bleeding for 2 months. Coronal enhanced MRI revealed a mass with irregular margins in the left ethmoid sinus that extended to the frontal base (Fig. 5a). Sagittal MRI showed that the tumor had invaded the frontal base bone and dura and was attached to the anterior wall of the sphenoid sinus (Fig. 5d). The patient underwent tumor resection. In addition to the removal of tumor, partial sphenoidotomy was performed to expose sufficient tumor margin for its total resection. The PNSF was used to cover the ASB defect through the bony support of the remnant sphenoid sinus wall. The pathological diagnosis was olfactory neuroblastoma. Postoperative coronal and sagittal MRI revealed a well enhanced PNSF and sufficient coverage of the ASB defect (Fig. 5b, e). The follow-up MRI taken 6 months after operation revealed no evidence of disease recurrence and good healing of nasal mucosa (Fig. 5c, f). There was no nasal complication and CSF leakage during the 9-months follow-up period.

#### Case 3: frontal base meningioma extended to the upper nasal cavity

**Fig. 6:**
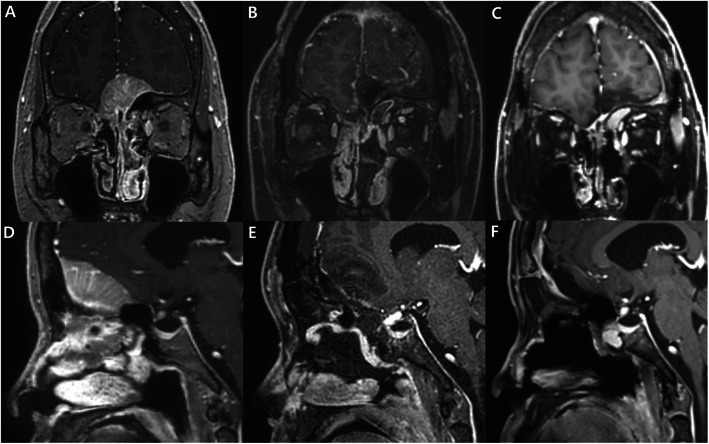
Images from Case 3. A meningothelial meningioma. **a** Coronal enhanced MRI reveals an extradural mass in the frontal base extending into the upper nasal cavity. **d** Sagittal MRI shows extensive involvement of the tumor in the upper nasal cavity, but not in the sphenoid sinus. **b**, **e** Postoperative contrast-enhanced coronal- and sagittal MRI shows a well-enhanced PNSF that covers the ASB defect. **c**, **f** The follow-up coronal and sagittal MRI showed small mucocele in the sphenoid sinus without any symptoms 6 months after the operation. ASB: anterior skull base, PNSF: pedicled nasoseptal flap

A 59-year-old woman presented with anosmia and headache. Coronal enhanced MRI revealed a mass in the frontal base that extended to the upper nasal cavity (Fig. 6a). Sagittal MRI showed extensive involvement of the tumor in the upper nasal cavity, but not in the sphenoid sinus (Fig. 6d). EEA and the transcranial approach were employed for the total removal of the tumor and ASB reconstruction. The posterior wall of the frontal sinus was also removed because of tumor involvement. The PNSF was used to cover the ASB defect, including the frontal sinus opening, and successful reconstruction was achieved without sphenoidotomy. We opened small part of the anterior sphenoid wall which was not covered by flap pedicle, to prevent future complications from sphenoid sinus obstruction. The pathologic diagnosis was meningothelial meningioma. Postoperative contrast-enhanced coronal and sagittal MRI showed that the PNSF completely covered the ASB defect with good enhancement (Fig. 6b, e). The follow-up MRI taken 6 months after operation showed small mucocele in the sphenoid sinus (Fig. 6c, f), but the patient has had no symptoms and discomfort.

## Discussion

The introduction of the PNSF by Hadad et al. [10], and the subsequent reduction in CSF leakage, has significantly improved the surgical outcomes of EEAs [11]. However, creating a PNSF of the correct size at the beginning of the surgery is important. Therefore, preoperative planning, including predicting the extent of the skull base defect accurately, and designing an adequately long PNSF for reconstruction, is critical. It is known to skull base surgeons that the length of the flap can be inadequate when ASB defect is reconstructed with routine PNSF. To identify this, there are some cadaveric and radioanatomic studies on PNSF and ASB reconstruction [1, 8, 9, 12–15]. However, in most of these studies, the ASB reconstruction was performed without sphenoidotomy, and therefore the findings cannot be applied to EEAs for large ASB lesions requiring sphenoidotomy [1, 9, 10, 13, 14]. In many cases, the ASB tumor is so large that it involves the planum sphenoidale and tuberculum sella, thereby requiring sphenoidotomy for its removal. In the present study, we evaluated the feasibility of using PNSF for ASB reconstruction, depending on whether sphenoidotomy was performed or not. While the PNSF provided coverage from the anterior wall of the sphenoid sinus to the posterior wall of the frontal sinus in all dissections without sphenoidotomy, it could not reach to the posterior wall of the frontal sinus in all dissections with sphenoidotomy. Ten Dam et al. in their study found that the PNSF was large enough to cover the skull base without sphenoidotomy but too small to cover the entire ASB after sphenoidotomy [15], which is consistent with our findings. The CL value was 1.86 ± 0.51 cm in the present study. The expected PNSF excess at the limbus of the sphenoid bone is important for the preoperative planning of EEAs to access the sella, tuberculum sella, and planum sphenoidale. In a Korean population study [8], the mean length of the planum sphenoidale was 1.26 ± 0.33 cm. Therefore, the PNSF could provide coverage up to around the planum sphenoidale in the EEAs with total sphenoidotomy considering the intraoperative shrinkage of the PNSF.

**Fig. 7:**
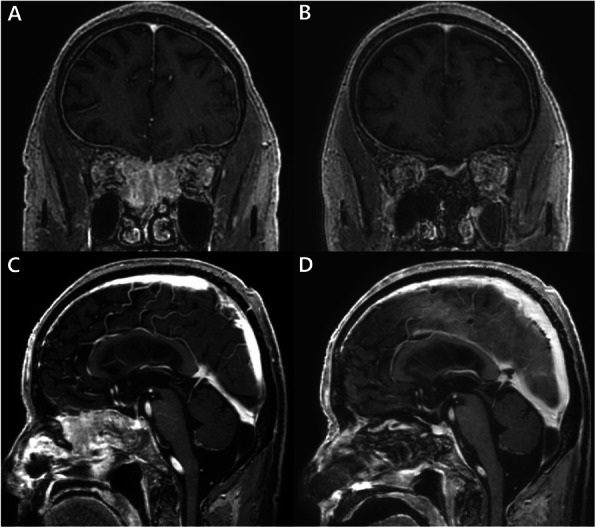
**a**, **c** A 78-year old male patient with huge olfactory neuroblastoma with extensive ASB defect which was revealed in preoperative enhanced coronal and sagittal MRI. **b**, **d** Postoperative coronal and sagittal MRI showed insufficient anterior coverage of PNSF for ASB defect, and loose attachment of PNSF to the skull base despite the support of tissue glue and nasal packing. The patient encountered the CSF rhinorrhea 3 days after the operation. ASB: anterior skull base, PNSF: pedicled nasoseptal flap

In case of a large lesion involving the entire ASB wherein sphenoidotomy is needed, other reconstruction techniques should be applied alone or in addition to PNSF. First, some studies have reported successful reconstruction using additional support like fat, artificial dura and glue. However, even this successful technique can result in the postoperative CSF leakage [16]. We also describe a case which showed the postoperative CSF leakage after placing the PNSF to the bony edge around the ASB using only additional support material without rigid bony structure to support it (Fig. 7). Second, multilayered reconstruction technique, pericranial flap, or several types of extended nasoseptal flaps can be solely applied to cover the large skull base defect [17–19]. Moreover, anteriorly based mucosal flaps, including anteriorly based inferior turbinate flap and anterior pedicle lateral nasal wall flap, can be added to cover the most anterior part of ASB, which cannot be covered by PNSF [20, 21]. To increase the anterior coverage of PNSF for lesions located most anterior part of ASB, modification technique using PNSF could be performed [6]. To make the choana incision along the wall over to the lateral nasal wall, the flap should be rotated up laterally so that the pedicle goes along the lamina, or the flap should be placed along the orbital apex and rotated into place while reducing the waste of length within the sphenoid sinus. These methods, though, provide better anterior coverage, but still run into the issue of not being able to get the flap much past the crista. Because of the inconsistency of these techniques, the anterior coverage in these methods could not be measured. In this study, we showed that the PNSF without sphenoidotomy could provide consistent anterior coverage by avoiding the pitfall of losing length in the sphenoid. In our three clinical cases, we did not perform total sphenoidotomy, because leaving the anterior wall of sphenoid sinus facilitated the PNSF to reach the posterior wall of the frontal sinus, based on our cadaveric results. We used the bony strut for a stable PNSF placement, and there was no postoperative CSF leakage even if perioperative lumbar drain was not used in any of the patients. However, our method could be applied to only selected ASB lesions, in which the tumor could be removed through EEAs with partial sphenoidotomy that leaves a remnant structure to support the PNSF.

An extra-long PNSF at the posterior wall of the frontal sinus indicates some degree of overlap in the margins. While a larger CPFS value clinically indicates a higher probability of successfully reconstructing the entire ASB defect, a smaller CPFS value indicates an increased risk of the PNSF length being insufficient. Our study showed successful coverage of the ASB over the posterior wall of the frontal sinus using PNSF in all patients without total sphenoidotomy. Some other studies have indicated that CPFS of ≤0.5 cm could be a risk factor for insufficient coverage for reconstruction [8, 9, 14]. In our cadaveric study without sphenoidotomy, the CPFS was ≤0.5 cm in 6 out of the 15 specimens (40%), unlike in some other studies that used Western cadavers and showed a lower rate of CPFS of ≤0.5 cm [7, 9]. These findings suggested that the degree of coverage in our study was relatively lower, which may have been due to a significantly shorter length of the PNSF in our Korean specimens than in the specimens of other studies [17]. Another radioanatomic study in a Korean population reported a CPFS of ≤0.5 cm in 29% of the patients [8]. We, however, found a slightly higher rate of PNSF insufficiency, possibly due to the use of cadavers in our study. This was because of the distortion of the flap arising from its rotation on the pedicle, resulting in its shortening or pulling. The actual dissections in our cadaveric study are a significant difference from the radioanatomic study.

This study has some limitations. First, we did not study the width of the ASB. The width of the PNSF is usually wide enough to cover the ASB defect, and the failure to cover the defect is usually due to the shortage of length. Additionally, methods of increasing the width of the PNSF has already been described in several studies, and extension of the inferior incision to the nasal floor can aid in obtaining a sufficient flap width to cover the ASB defect [5, 22, 23]. Therefore, this study focused on the length of the PNSF. Second is the small sample size. It is difficult to extrapolate the results from 15 specimens to whole populations. However, Korea has a single ethnic group with relatively similar characteristics. Moreover, compared to some other cadaveric studies [12, 15, 24, 25], the number of specimens in the present study is not small. Third, while the method using PNSF without sphenoidotomy can be applied in cases of anteriorly located tumor in the ASB by increasing the anterior coverage, it cannot be the solution for cases with large tumors in the ASB. In such cases, sphenoidotomy may be essential to remove the tumors totally, and the skull base defects could include the planum or tuberculum, and therefore, this technique may not be adequate. However, because this technique can be applied by leaving only some portion of the anterior sphenoid wall for supporting the PNSF, it can help with the successful reconstruction in some cases with tumors in the ASB. Fourth, there were some discrepancies between the cadaveric and clinical studies. After total sphenoidotomy, there were some concerns about whether the PNSF needs to be completely placed in the sphenoid sinus wall. In our cadaveric study, we performed the reconstruction for comparison group which the PNSF was completely placed in the entire contour of the sphenoid sinus wall to obtain a reliable measurement of the length. With these results we can suggest the shortage of flap coverage for large ASB defect using conventional PNSF, and the advantage of covering the PNSF in length when leaving the anterior wall of the sphenoid sinus.

## Conclusions

Without sphenoidotomy, the PNSF could reach the posterior wall of the frontal sinus, making it possible to reconstruct the ASB defect successfully. However, in the case of a large ASB tumor, total sphenoidotomy might be needed for tumor removal, and additional reconstruction methods may also be required to cover a large ASB defect. If possible, by leaving the lateral side of the anterior sphenoid wall for supporting the PNSF, successful ASB reconstruction could be achieved following the endoscopic resection of ASB tumors. The results of this cadaveric study and our clinical cases indicate that preoperative planning of EEAs, including sphenoidotomy, is essential to reduce postoperative CSF leakage.

## Data Availability

The datasets used and/or analysed during the current study are available from the corresponding author on reasonable request.
